# Analysis of the Potential Distribution of *Solanum rostratum* in China Based on the Biomod2 Ensemble Model

**DOI:** 10.3390/plants15050816

**Published:** 2026-03-06

**Authors:** Yue Zhang, Weige Ma, Quanlai Zhou, Wei Cao, Bo Qu, Jia Guo, Li Zhou, Jiaojiao Deng, Yansong Zhang, Yanan Li, Limin Dai

**Affiliations:** 1 CAS Key Laboratory of Forest Ecology and Silviculture, Institute of Applied Ecology, Chinese Academy of Sciences, Shenyang 110016, China; 2 Key Laboratory of Global Change and Biological Invasion of Liaoning Province, College of Biological Science and Technology, Shenyang Agricultural University, Shenyang 110866, China

**Keywords:** invasive alien plant, *Solanum rostratum*, environmental variables, Biomod2 ensemble models, potentially suitable areas

## Abstract

*Solanum rostratum* is a globally regulated invasive species, known for its detrimental impacts on local biodiversity, human and livestock health, and agricultural productivity. This study employed the Biomod2 ensemble modeling framework to analyze the geographic distribution of *S. rostratum* in China, identify key environmental factors limiting its spread, and provide a scientific basis for its management and control. By integrating species distribution data with multiple environmental variables, we predicted the potential geographic distribution of this species. Pearson correlation analysis and variance inflation factor (VIF) testing were applied to identify significant environmental variables constraining its spread, including precipitation seasonality (bio15), mean temperature of the wettest quarter (bio8), precipitation of the warmest quarter (bio18), isothermality (bio3), precipitation of the driest month (bio14), and human footprint. Three Biomod2-based ensemble models (EMmean, EMca and EMwmean) were based on the receiver operating characteristic curve (ROC), true skill statistic (TSS), and Kappa coefficient. Of these, EMca demonstrated the highest predictive accuracy. The model identified highly suitable habitats for *S. rostratum* primarily in semi-arid and semi-humid regions with high human activity, including the Northeast Plain, bounded by the Greater Khingan, Lesser Khingan, and Changbai Mountains; the northern North China Plain extending to the Shandong Hills and Yellow River basin; and the Junggar Basin extending to the Altai Mountains. These regions should be prioritized for future monitoring and control efforts. This study provides both empirical data and theoretical insights to accurately delineate potential invasion zones of *S. rostratum*, enhancing surveillance and guiding effective prevention and control strategies.

## 1. Introduction

*Solanum rostratum* Dunal (Solanaceae) is a globally regulated quarantine pest, widely recognized as a major invasive species [1]. Native to Mexico and the United States [2], it has expanded its range as a non-native invasive species to North America, Asia, Europe, South Africa, and Oceania [3]. The species was first recorded in China in 1982, in Chaoyang City, Liaoning Province [4]. In recent years, it has spread to twelve provincial-level administrative regions, including Heilongjiang, Jilin, Liaoning, Inner Mongolia, Hebei, Beijing, Tianjin, Shandong, Shanxi, Gansu, Ningxia Hui Autonomous Region, and Xinjiang Uyghur Autonomous Region. This invasion has led to biodiversity loss, posed threats to human and livestock health, and reduced crop yields, severely impacting China’s ecological environment. In December 2016, *S. rostratum* was officially included in the “Fourth Batch of Invasive Alien Species List” issued by the Ministry of Ecology and Environment of the People’s Republic of China [5]. It was subsequently added to the “Catalogue of Plant Quarantine Pests Imported into the People’s Republic of China” in June 2017 [6]. Recent studies indicate that the species spreads at an approximate rate of 16 km per year [7], prompting a need for accurate predictions of its potentially suitable areas to inform targeted prevention and control strategies.

The prediction of suitable areas for alien invasive plants is primarily achieved through species distribution models (SDMs). These models are constructed using known species occurrence data, which are then linked to environmental gradients to predict potentially suitable areas [8]. Over recent decades, SDMs have advanced significantly, with models generally categorized into single models and ensemble models. Common single-model approaches include Maximum Entropy Modeling (MaxEnt), Classification Tree Analysis (CTA), Flexible Discriminant Analysis (FDA), Generalized Additive Models (GAMs), Gradient Boosting Models (GBMs), Generalized Linear Models (GLMs), Multivariate Adaptive Regression Splines (MARSs), Random Forest (RF), Surface Range Envelope (SRE), and Extreme Gradient Boosting (XGBoost), among others [9,10,11]. Single models offer advantages such as simplicity, ease of implementation, intuitive and easily interpretable outputs, high flexibility, adaptability, and computational efficiency [12]. However, they also have notable limitations of single models, including susceptibility to overfitting, challenges in modeling complex nonlinear relationships, and restricted interpretability of predictive results [13,14,15]. To overcome these limitations while retaining the advantages of single models, recent studies have increasingly employed the Biomod2 ensemble modeling framework.

The Biomod2 ensemble framework simultaneously executes multiple single models, such as CTA, FDA, GAM, GBM, GLM, MARS, MaxEnt, RF, SRE, and XGBoost, and integrates their outputs. This approach effectively reduces the high variance typical of complex single models and mitigates the high bias often observed in simpler models. Additionally, it provides a standardized and reproducible workflow, ensuring high accuracy and reproducibility of research findings [16,17,18,19,20]. Thus, Biomod2 has become comprehensive and reliable for predicting potentially suitable areas of invasive plant species.

Traditionally, climatic variables have been identified as the primary determinants of species distribution, and they have been the main predictors in SDMs for alien invasive plants [14,21,22,23]. However, recent studies suggest that the distribution of these species results from a combination of climate, soil, topography, and anthropogenic factors [17]. In support of this, several studies have incorporated climatic, edaphic, topographic, and anthropogenic factors into species distribution models to improve their predictive accuracy [24,25,26]. Whether climatic variables alone can provide an accurate prediction of alien invasive plant distributions or if a multifactorial approach integrating climate, soil, topography, and human activity yields more precise predictions remains a critical issue warranting further investigation.

In this study, distribution data for *S. rostratum* in China were compiled from multiple sources to map its geographic distribution. A correlation analysis of environmental and anthropogenic variables was conducted to identify the key factors influencing this species’ distribution. Subsequently, an optimal Biomod2 ensemble model was developed to predict the potentially suitable areas of *S. rostratum* across China. This research provides a case study demonstrating the application of the Biomod2 ensemble framework for predicting potentially suitable areas of alien invasive plants and provides technical support for early-warning systems and monitoring of invasive species.

## 2. Results

### 2.1. Distribution of Solanum rostratum in China

**Figure 1:**
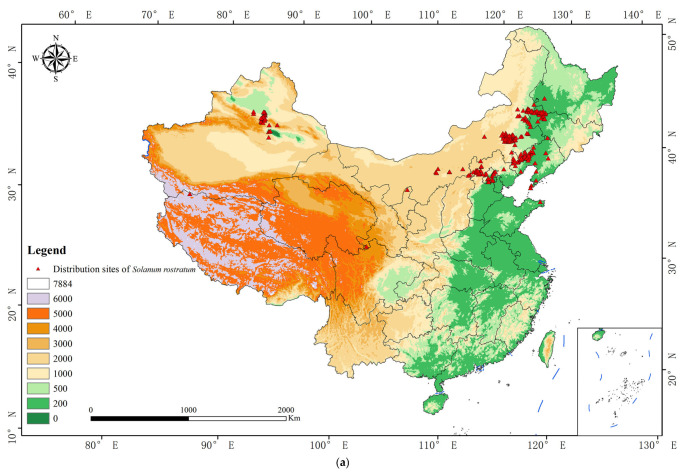

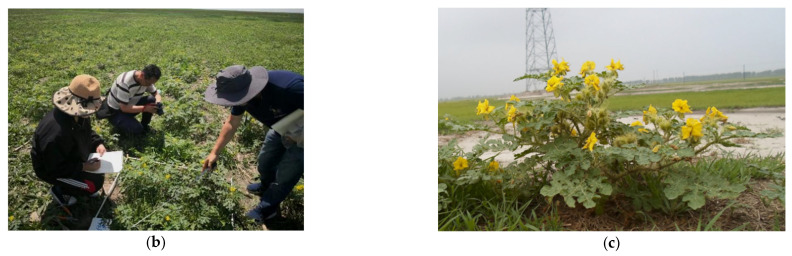
(**a**) Distribution map of *S. rostratum* in China. (**b**) Field research on *S. rostratum*. (**c**) Field photograph of *S. rostratum*.

A distribution map of *S. rostratum* in China was generated based on its occurrence points (Figure 1). The results revealed that *S. rostratum* is predominantly found in twelve provincial-level regions across northern China, including Heilongjiang, Jilin, Liaoning, Inner Mongolia, Hebei Province, Beijing Municipality, Tianjin Municipality, Shandong Province, Shanxi, Gansu Province, Ningxia Hui Autonomous Region, and Xinjiang Uygur Autonomous Region. Approximately 90% of the occurrence points were located at elevations below 1000 m, while the remaining points were distributed between 1000 and 3500 m. As shown in Figure 1, the species primarily inhabits three regions: the Northeast Plain, the northern North China Plain, and the foothills of the Tianshan Mountains in Xinjiang. These regions were identified as key targets for monitoring and control efforts.

### 2.2. Selection of Influencing Variables for Modeling

**Figure 2:**
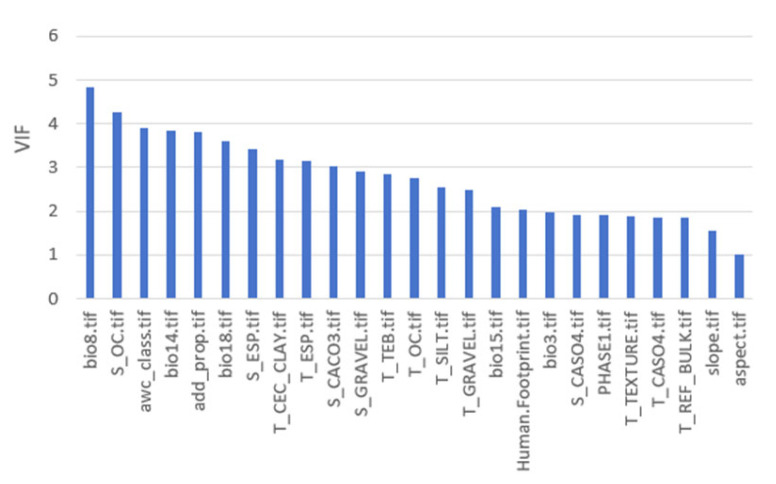
Variance inflation factor (VIF) values of environmental variables used in species distribution models.

**Figure 3:**
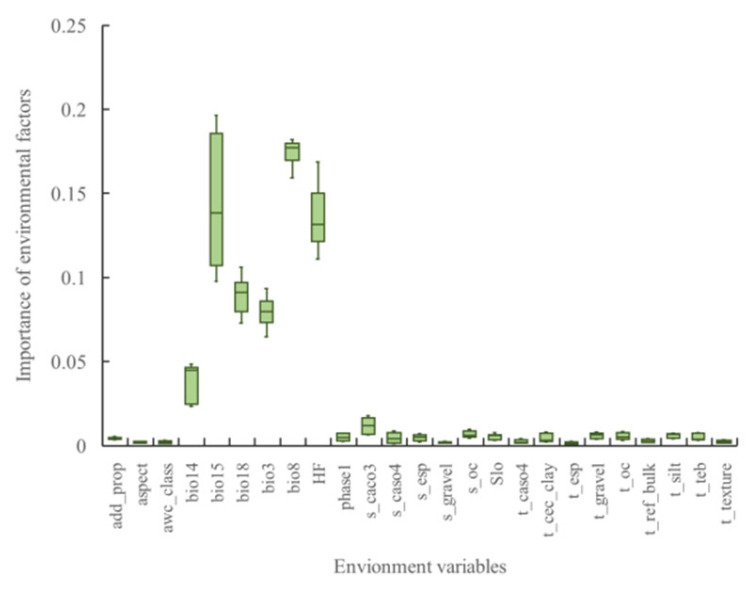
Relative importance scores of environmental variables influencing the models.

This study selected 25 environmental variables with variance inflation factor (VIF) values below five (Figure 2). The relative importance of these factors is shown in Figure 3. The analysis identified six ecological variables as having the greatest influence on the invasion of *S. rostratum*: precipitation seasonality (bio15), mean temperature of the wettest quarter (bio8), human footprint (HF), precipitation of the warmest quarter (bio18), isothermality (bio3), and precipitation of the driest month (bio14). These six variables accounted for 92.3% of the total relative importance, with five hydrothermal climatic variables contributing 74.4% and the anthropogenic factor (human footprint) accounting for 17.6% of the total. The remaining 19 variables collectively accounted for only 7.7% of the overall importance.

### 2.3. Analysis of Major Influencing Variables

**Figure 4:**
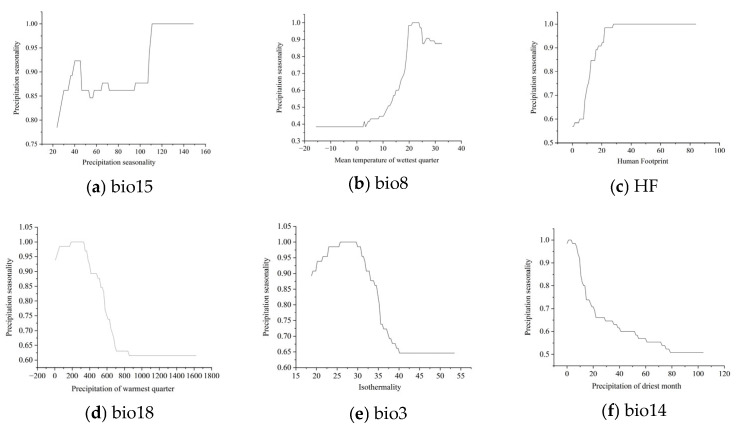
Response curves of six key environmental variables.

The response curves for the six key influencing variables are shown in Figure 4. The findings can be summarized as follows:

(1) Precipitation seasonality (Bio15): A value of 117 or higher indicated regions with pronounced seasonal precipitation variation, promoting favorable growth conditions for *S. rostratum*. The species thrived in areas with significant monthly fluctuations in precipitation, growing rapidly during the rainy season and surviving drought through seed dormancy.
(2) Mean temperature of the wettest quarter (Bio8): A range of 20 °C to 25 °C supports optimal growth conditions for *S. rostratum*, providing a warm but not excessively hot climate.
(3) Human footprint (HF): Areas with HF values exceeding 29, typically urban outskirts, transportation corridors, and agricultural land, facilitated the spread of *S. rostratum*.
(4) Precipitation of the warmest quarter (Bio18): A total rainfall of between 200 mm and 400 mm during the hottest three months ensures the species’ vigor, mitigating drought stress and preventing disease outbreaks.
(5) Isothermality (Bio3): Values between 25 °C and 32 °C indicated moderate temperature stability without extreme seasonal cold, promoting growth.
(6) Precipitation of the driest month (Bio14): Precipitation in the range of 0 to 80 mm was related to *S. rostratum* survival. Higher precipitation within this range reduced survival, stabilizing at approximately 50% when it exceeded 80 mm.

These findings suggest that semi-arid to semi-humid regions with warm climates, absence of extreme low-temperature stress, and frequent human activities constitute optimal conditions for the rapid proliferation of *S. rostratum*. These areas should be prioritized for strategies to prevent and control their spread.

### 2.4. Development and Evaluation of the Biomod2 Ensemble Model

**Figure 5:**
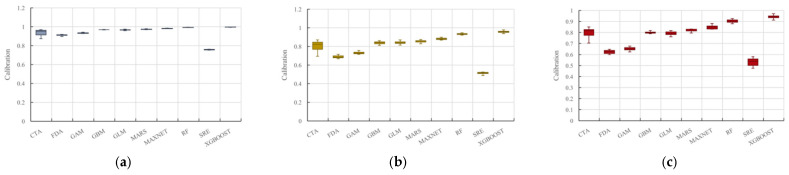
Model accuracy evaluations using AUC, TSS, and Kappa metrics: (**a**) AUC; (**b**) TSS; (**c**) Kappa.

This study evaluated ten individual models—CTA, FDA, GAM, GBM, GLM, MARS, MAXENT, RF, SRE, and XGBOOST. Eight models (CTA, GAM, GBM, GLM, MARS, MAXENT, RF, and XGBOOST) met the performance thresholds of AUC > 0.9, TSS > 0.8, and Kappa > 0.7 (Figure 5).

**Figure 6:**
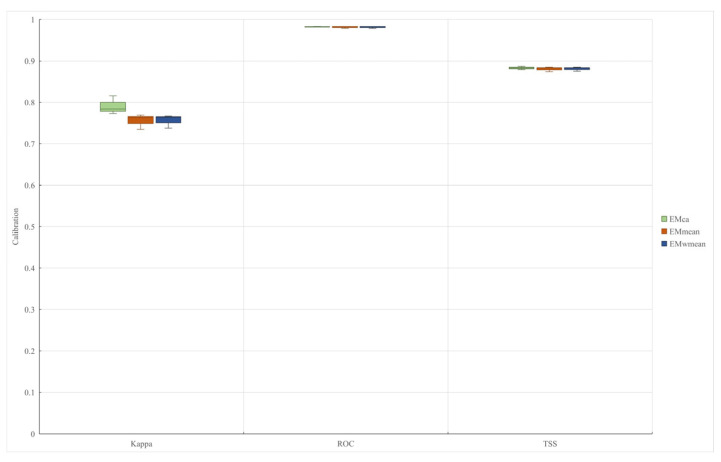
Accuracy assessment of three integrated models.

Based on the eight individual models derived from Biomod2, three ensemble models —EMmean, EMca, and EMwmean—were constructed and evaluated (Figure 6). All three models met the performance criteria of AUC > 0.9, TSS > 0.8, and Kappa > 0.7, indicating their suitability for analyzing potentially suitable areas of *S. rostratum* in China. Among them, EMca exhibited the highest predictive accuracy (AUC = 0.9838, TSS = 0.887, Kappa = 0.816) and was, therefore, selected as the optimal ensemble model. Consequently, the EMca model was used to predict the potential distribution of *S. rostratum* across China.

### 2.5. Potential Distribution of S. rostratum in China

**Figure 7:**
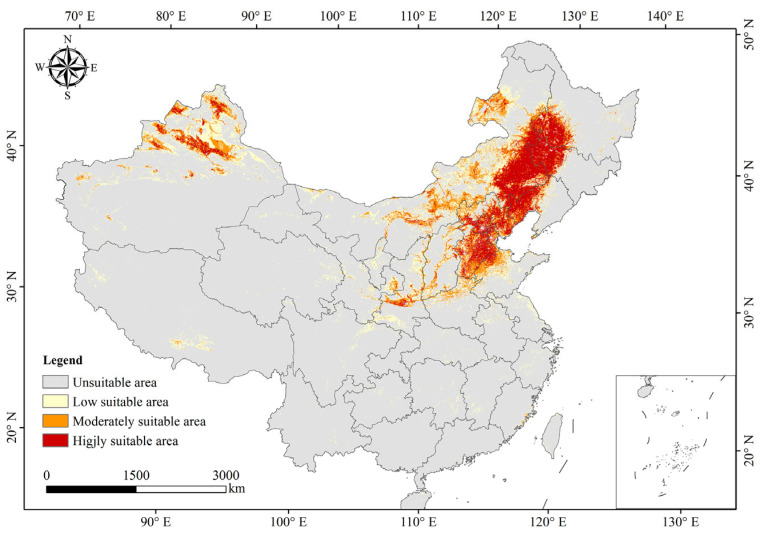
Potentially suitable areas of *S. rostratum* in China.

The optimal ensemble model (EMca) derived from Biomod2 was used to predict the potentially suitable areas of *S. rostratum* in China (Figure 7; Table 1). The results showed the following:

(1) The total potential suitable area for *S. rostratum* was estimated at 162.65 × 10^4^ km^2^, accounting for 16.94% of China’s land area. This area was primarily concentrated in the Inner Mongolia Autonomous Region, Heilongjiang, Jilin, Liaoning, Hebei, Beijing, Tianjin, Shandong, Shanxi, Shaanxi, Henan, Gansu, and Xinjiang. Except for the Macao Special Administrative Region, occurrences were observed in all other provincial regions.
(2) The highly suitable area covered approximately 44.63 × 10^4^ km^2^, representing 4.65% of the national land area. It was concentrated in the eastern and central parts of Inner Mongolia, western Heilongjiang, northwestern Jilin, northwestern Liaoning, Hebei, Beijing, Tianjin, Shandong, Shanxi, Shaanxi, and northern Xinjiang, with scattered occurrences in Henan and Gansu.
(3) The low suitability area encompassed about 73.02 × 10^4^ km^2^, accounting for 7.61% of the national land area. It was mostly distributed in Inner Mongolia, eastern and western Heilongjiang, central Jilin, central Liaoning, Hebei, Beijing, Tianjin, Shanxi, Shaanxie, Shandong, and northern Xinjiang, with scattered presence across other regions except Macao.

**Table 1:** Potentially suitable areas of *S. rostratum*.

Types of Suitable Areas	Acreage of Suitable Areas (km^2^)	Percentage of the Total National Area
Highly suitable area	44.63347 × 10^4^	4.65%
Moderate suitable area	44.99792 × 10^4^	4.68%
Low suitable area	73.02111 × 10^4^	7.61%
total	162.6525 × 10^4^	16.94%

Figure 7 illustrates that the highly suitable areas for *S. rostratum* are distributed across 13 provincial regions, including Inner Mongolia, Heilongjiang, Jilin, and Liaoning. These areas predominantly fall within China’s semi-arid to semi-humid climatic zones. Spatially, highly suitable areas are primarily concentrated in three distinct regions: (1) the Northeast Plain, bounded by the Greater Khingan, Lesser Khingan, and Changbai Mountains; (2) the northern North China Plain extending to the Shandong Hills and along the Yellow River; and (3) the corridor from the Junggar Basin to the Altai Mountains.

## 3. Discussion

### 3.1. Influence of Environmental Variables on Species Distribution

Early studies on species distribution primarily relied on climatic variables to model range shifts [7,17,27,28,29]. These approaches defined a species’ fundamental ecological niche as the range of environmental variables it can physiologically tolerate. The availability of comprehensive, data-rich climate databases (e.g., WorldClim and CHELSA) made these models especially suitable for studying climate-change-driven changes in species distributions on global and continental scales. However, with methodological advancements, additional variables—such as topography, soil properties, and anthropogenic influences—have increasingly been integrated into distribution models. Recent studies have demonstrated that relying solely on climatic variables can lead to overestimations of suitable areas and overlook local-scale heterogeneity, resulting in less precise predictions that are unsuitable for site-specific management [12,29]. Integrating multiple environmental variables enables the capture of local distributional nuances unexplained by climate alone, improving model accuracy and facilitating the identification of key limiting variables beyond climate [30]. For example, research on invasive alien plants in Northeast China highlighted human population density and proximity to ports as critical factors in species distributions [31]. Similarly, the distribution of the Asian clam (*Corbicula fluminea*) was found to be influenced by both temperature and elevation, in addition to annual mean temperature and the coldest quarter’s temperature [32]. Likewise, research on *S. rostratum* in Xinjiang revealed that, beyond hydrological and thermal factors, intensity and elevation of human activity substantially affected its spatial distribution [33].

This study revealed that *S. rostratum* exhibits optimal growth conditions in invaded areas characterized by a mean temperature of the wettest quarter (bio8) ranging from 20 °C to 25 °C, precipitation of the warmest quarter (bio18) between 200 mm and 400 mm, and precipitation of the driest month (bio14) between 0 and 80 mm. Bio14 represents precipitation of the driest month, capturing minimum water availability during the most limiting period of the year; for many herbaceous and invasive plant species, establishment and survival are strongly constrained by drought stress during this critical window [34,35]. The stabilization of predicted suitability above approximately 80 mm suggests a threshold-type response: once a minimum moisture requirement is met, additional precipitation in the driest month contributes limited marginal gains to survival probability, consistent with the notion that water limitation can act as a primary environmental filter, the effect of which diminishes after physiological demands are satisfied [36]; empirical SDM evidence shows strong sensitivity to minimum/seasonal water constraints and related drought metrics [37,38].

These conditions jointly indicate pronounced precipitation seasonality, moderate temperature regimes without extreme seasonal cold, and frequent human activities. Such environmental parameters correspond to the hydrothermal climate typical of China’s semi-arid to semi-humid regions [39]. The highly suitable areas for *S. rostratum* were primarily distributed in eastern Inner Mongolia, the southwestern Songnen Plain, the northern North China Plain, and northern Xinjiang, largely confined within China’s semi-arid to semi-humid zones. Accordingly, based on the environmental and anthropogenic variables identified, it is concluded that areas within these climatic zones with relatively high levels of human activity constitute the optimal ecological niche for *S. rostratum* distribution and are susceptible to rapid population expansion. Consequently, future monitoring and management efforts should prioritize these regions to effectively control the invasion of *S. rostratum.*

### 3.2. Dispersal Mechanisms and Human-Mediated Spread

Solanum rostratum exhibits several life-history traits associated with the success of invasion, including prolific seed production, high seed viability, and the capacity to form persistent soil seed banks. Such traits enhance colonization probability once propagules reach suitable habitats. The species produces spiny burs that readily attach to animals and agricultural equipment, facilitating short- to medium-distance dispersal. Natural spread likely occurs through gravity, wind-assisted rolling of senescent plants, and epizoochory. However, these mechanisms primarily operate at local scales and cannot fully explain rapid regional expansion. Similar trait–invasion relationships have been widely documented in annual invasive plants [36,40].

Our model revealed a substantial contribution of anthropogenic variables, highlighting the pivotal role of human activities in shaping the current and potential distribution of *S. rostratum*. Road density, land-use intensity, and human footprint indices were strongly associated with habitat suitability. Transportation networks function as dispersal corridors, increasing connectivity among suitable habitats and elevating propagule pressure [41,42]. In agricultural systems, dispersal may occur via contaminated crop seeds, soil movement, livestock transport, and transfer of machinery. The adhesive morphology of the fruit enhances accidental long-distance transport, accelerating secondary spread.

These findings align with global evidence indicating that human-mediated dispersal has become the dominant driver of plant invasions under increasing globalization and land-use change [43,44]. Thus, while climate defines a potentially niche space, anthropogenic processes largely determine invasion realization and spatial expansion rates.

Recognizing dispersal pathways provides a mechanistic basis for prevention strategies. Management efforts should prioritize (1) strengthened quarantine and seed inspection systems, (2) mandatory sanitation of agricultural machinery, (3) monitoring of high-risk transportation corridors, and (4) early detection and rapid response in climatically suitable yet currently uninvaded regions. Integrating species distribution modeling with pathway analysis enhances proactive invasion management and improves early-warning frameworks [18,45].

### 3.3. Analysis of Biomod2 Ensemble Model

Compared with earlier studies that primarily relied on a single algorithm, such as MaxEnt, this study adopted a multi-algorithm ensemble SDM framework implemented in biomod2, which helps quantify inter-algorithm uncertainty and reduces reliance on algorithm-specific assumptions [12,39,46,47,48]. In addition, we incorporated a broader set of predictors (climate, soil, topography, and anthropogenic variables), enabling a more mechanistic and ecologically integrative interpretation of the distribution patterns and improving model transferability when environmental covariates are sampled unevenly [16,49,50]. Model performance was evaluated consistently across algorithms using multiple metrics (e.g., ROC and TSS), and ensemble predictions were constructed based on model performance, thereby improving the robustness of suitability estimates relative to single-model outputs [51,52].

Within biomod2, three ensemble strategies are most commonly applied: EMmean, EMwmean, and EMca [52]. EMmean (simple averaging) is computationally efficient and tends to be less sensitive to extreme predictions, thus providing smooth suitability gradients; however, equal weighting may dilute the contribution of the best-performing algorithms. EMwmean addresses this limitation by weighting model predictions using evaluation scores, typically increasing overall accuracy by emphasizing stronger models; nevertheless, if the chosen metrics do not reflect true generalization, weighting can inflate apparent validation performance and increase overfitting risk, particularly under spatially structured sampling [47,49]. EMca (committee averaging/majority consensus) is more conservative, prioritizing agreement among algorithms; this often yields more stable and policy-relevant maps for invasive species screening by reducing sensitivity to any single model’s idiosyncrasies [48]. In our case, all three ensemble methods performed well for forecasting potentially suitable areas of *S. rostratum*, but EMca achieved the highest evaluation scores, indicating that consensus-based integration most effectively delineated the potential distribution of *S. rostratum* in China.

### 3.4. Analysis of the Potential Distribution of Solanum rostratum in China

Only a limited number of studies have investigated the potentially suitable areas of *S. rostratum* invading China [7,26,29,33,53]. These studies, primarily focused on Northeast China, identified that highly suitable areas were mainly distributed in western and southern Liaoning Province as well as the southeastern Inner Mongolia Autonomous Region, with suitability levels gradually declining radially outward from these core zones [7]. The findings of the present study are consistent with these earlier results. Research conducted in Xinjiang demonstrated that *S. rostratum* has been expanding radially from Changji Prefecture and Urumqi City toward regions north of the Tianshan Mountains and the western parts of Xinjiang [33], closely aligning with the outcomes of this study. More extensive investigations across China have shown that suitable areas for *S. rostratum* are predominantly concentrated in the northeastern and northwestern regions [53], with the North China Plain identified as a high-risk zone for potential spread [29]. Projections further indicate that the principal distribution of *S. rostratum* in China will remain concentrated in northern China [26]. Building upon prior research, this study more precisely delineated the potentially suitable areas of *S. rostratum* as regions within northern China’s semi-arid to semi-humid zones characterized by relatively frequent human activities. These regions span thirteen provincial-level administrative units, including the Inner Mongolia Autonomous Region, Heilongjiang Province, Jilin Province, and Liaoning Province. By enhancing the spatial resolution and accuracy of area suitability predictions, the present study effectively characterizes the distribution of this species within heterogeneous environments and anthropogenically influenced areas, thereby providing a more realistic and detailed projection of its potentially suitable range.

In highly suitable regions of China, *S. rostratum* should be surveyed at least twice annually (early growth and pre-seeding), with additional monthly checks along roadsides, construction sites, croplands, and transport hubs. UAV/satellite screening can be combined with GPS-referenced transects and quadrat searches to map infestations and detect recruits. Efforts should be standardized, geotagged photos archived, and report records stored on a centralized database to trigger rapid response and follow-up inspections within 4–6 weeks after removal.

## 4. Materials and Methods

### 4.1. Distribution Data

Species distribution data for *S. rostratum* were compiled from four primary sources: (1) occurrence records from the Global Biodiversity Information Facility (GBIF.org, GBIF Occurrence Download https://doi.org/10.15468/dl.8yxaze (accessed on 16 September 2024)); (2) specimen records from the National Specimen Information Infrastructure (NSII.org, https://www.nsii.org.cn/2017/query.php?name=%27%27 (accessed on 10 July 2025)) in China; (3) information extracted from published journal articles and books; and (4) field survey data collected by the research team at known distribution sites. In total, 377 occurrence records within China were assembled. To ensure compatibility with environmental predictors and reduce spatial sampling bias, duplicate records within the 30-arcsecond grid were removed by retaining a single occurrence per grid cell. Potential spatial autocorrelation was further minimized via spatial thinning using a 1 km minimum nearest-neighbor distance, implemented with the spThin package (version 0.1.0) in R. After filtering, 283 occurrence points remained (Figure 1). To enhance transparency, a map showing the spatial distribution of the species is provided; this figure illustrates the reduction in spatial clustering following filtering.

### 4.2. Environmental Variables

This study compiled 59 environmental variables, encompassing 19 climatic, 35 soil, 3 topographic, and 2 anthropogenic variables (Table 1). Climatic variables were obtained from the WorldClim database (WorldClim.org, https://www.worldclim.org/data/worldclim21.html (accessed on 25 July 2025)) at a spatial resolution of 30 arc-seconds for the baseline period of 1970–2000. Soil variables were derived from the World Soil Database v2.0 available via the FAO Soil Portal (FAO.org, https://www.fao.org/statistics/data-collection/general/en (accessed on 28 July 2025)) and also at 30-arcseconds. Topographic variables (elevation, slope, and aspect) were extracted from the International Scientific Data repository of the Computer Network Information Center, Chinese Academy of Sciences (GSCloud.cn, http://www.gscloud.cn/sources/index?pid=1&rootid=1 (accessed on 29 July 2025)) with a spatial resolution of 25 m. Anthropogenic variables, including the Human Influence Index (HII) and Human Footprint (HF), were obtained from the Center for International Earth Science Information Network (CIESIN), Columbia University (CIESIN Columbia University.org, https://ciesin.columbia.edu/content/data (accessed on 28 July 2025)). To ensure spatial consistency, all datasets were projected to a common coordinate reference system (GCS_WGS_1984), resampled to a uniform spatial resolution of 30 arc-seconds, and aligned to a common grid prior to analysis. The HF dataset was resampled using bilinear interpolation.

Prior to modeling, multicollinearity among predictors was assessed using Pearson correlation analysis and variance inflation factors (VIFs). The correlation between HF and elevation was moderate and negative (r = −0.57), indicating no strong linear dependence. Variable selection was further guided by the Akaike Information Criterion (AIC), and only predictors with VIF < 5 were retained to avoid problematic multicollinearity [54,55]. Ultimately, 25 variables met the selection criteria, including 5 climatic, 17 soil, 2 topographic, and 1 anthropogenic predictor (Table 2).

### 4.3. Construction and Evaluation of the Biomod2 Ensemble Models

Modeling was conducted using the biomod2 package (version 4.2-3) in R 4.3.1. Species occurrence records and environmental predictors were formatted using the biomod_formating function, and 30,000 pseudo-absence points were randomly generated to calibrate the models. Model parameters were optimized with the biomod_tuning function prior to model fitting. For each modeling iteration, 75% of the dataset (including presence and pseudo-absence records) was randomly selected for training, while the remaining 25% was used for model evaluation. Occurrence and pseudo-absence data were assigned equal weights. The modeling procedure was repeated five times to ensure robustness. Individual models were evaluated using three performance metrics: the area under the receiver operating characteristic curve (ROC), the true skill statistic (TSS), and Cohen’s Kappa. Based on the selected high-performing models, three ensemble approaches were implemented in biomod2: Ensemble Mean (EMmean), committee averaging (EMca), and Weighted Mean (EMwmean). The predictive performance and stability of the ensemble models were assessed using ROC, TSS, and Kappa statistics. The ensemble model with the highest predictive accuracy and lowest uncertainty was selected as the optimal model and subsequently used to project the potentially suitable distribution of *S. rostratum*.

### 4.4. Delineation of Potentially Suitable Areas

The Jenks natural breaks classification method was applied to categorize suitable areas predicted by the model [56].

## 5. Conclusions

*Solanum rostratum* has invaded twelve provincial-level regions in China, with approximately 90% of its documented occurrence points situated at elevations below 1000 m in northern China. The primary invasion areas include the Northeast Plain, the northern North China Plain, and the foothills of the Tianshan Mountains in Xinjiang. Among the four categories of influencing variables—climatic, edaphic (soil), topographic, and anthropogenic—climatic and human activity variables were identified as the most critical determinants of *S. rostratum*’s distribution within China. Specifically, five climatic variables exerted the greatest influence on their distribution: precipitation seasonality (bio15), mean temperature of the wettest quarter (bio8), precipitation of the warmest quarter (bio18), isothermality (bio3), and precipitation of the driest month (bio14). These five variables together account for 74.4% of the total variable importance. Human activity variables were the next most influential, contributing 17.6% to the model’s explanatory power. Using the Biomod2 ensemble modeling framework, the committee averaging (EMca) model was identified as an optimal predictor of *S. rostratum*’s suitable areas in China. Model projections revealed that highly suitable areas are concentrated in semi-arid to semi-humid regions with frequent human activities across thirteen provincial-level administrative regions, including Inner Mongolia, Heilongjiang, Jilin, and Liaoning. These highly suitable areas were primarily divided into three regions: (1) the Northeast Plain, bounded by the Greater Khingan Range, Lesser Khingan Range, and Changbai Mountains; (2) the northern North China Plain, extending to the Shandong Hills and along the Yellow River; and (3) the area spanning from the Junggar Basin to the Altai Mountains.

## Figures and Tables

**Table 2:** Environmental variables employed in the modeling framework of this study.

Type	Abbreviation	Description
Climatic Variables	Bio3	Isothermality
	Bio8	Mean temperature of wettest quarter (°C)
	Bio14	Precipitation of driest month (mm)
	Bio15	Precipitation seasonality (mm)
	Bio18	Precipitation of warmest quarter (mm)
Soil Variables	AWC_CLASS	Available water content of soil (%)
	PHASE1	Soil phase
	ADD_PROP	Specific soil types related to agricultural use in soil units
	T_GRAVEL	Topsoil gravel content
	T_SILT	Topsoil silt fraction (%)
	T_REF_BULK	Topsoil reference bulk density
	T_OC	Topsoil organic carbon content (%)
	T_CEC_CLAY	Topsoil CEC (clay)
	T_TEB	Topsoil TEB
	T_CASO4	Topsoil calcium sulfate content
	T_ESP	Topsoil sodicity (ESP)
	T_TEXTURE	Topsoil texture
	S_GRAVEL	Lower soil gravel content
	S_OC	Lower soil organic carbon content (%)
	S_CACO3	Lower soil calcium carbonate content
	S_CASO4	Lower soil calcium sulfate content
	S_ESP	Lower soil sodicity (ESP)
Topographic Variables	Slo	Slope (°)
	Asp	Aspect (°)
Anthropogenic Variables	HF	Human footprint

## Data Availability

The original contributions presented in this study are included in the article. Further inquiries can be directed to the corresponding author.
